# Enriching Psychological Research by Exploring the Source and Nature of Noise

**DOI:** 10.1177/17456916241235889

**Published:** 2025-03-04

**Authors:** Joakim Sundh, Philip Millroth, August Collsiöö, Peter Juslin

**Affiliations:** Department of Psychology, Uppsala University

**Keywords:** exogenous versus endogenous, methodology, noise, statistical modeling

## Abstract

In psychological research, noise is often considered a nuisance that obscures rather than contributes information. This simplification overlooks that noise can be informative and that by exploring the nature of the noise one can often draw additional conclusions concerning the underlying psychological processes. It is arguably only in recent years that the mainstream of researchers has taken this idea to heart and demonstrated that it can lead to breakthroughs in the understanding of human behavior. The aim of this special section is to showcase some of the ways in which systematic exploration of noise can be achieved and how it can enrich psychological research. In this introductory article, we introduce the idea of treating noise as endogenous as opposed to exogenous to the theoretical and statistical models of psychological phenomena. We then contribute a historical review of the role of noise in psychological research, including discussions of previous endogenous treatments of noise in the literature. As an illustration, we describe our own research on the precise/not precise model and show how noise distributions can be used to delineate analytic and intuitive modes of reasoning. Finally, we briefly introduce the other contributions to this special section.

Noise is a ubiquitous phenomenon. Practically all areas of human behavior and human experience involve some measure of unpredictable variability. In psychological research in general and in statistical modeling in particular, noise is most often viewed as something undesirable, a nuisance that obscures information about the phenomenon under study rather than an integral and potentially informative part of the phenomenon. In terms of statistical methodology, noise is therefore generally conceptualized as a separate term added to an otherwise deterministic model to describe unexplained variability in data.^1^ Although it has often been noted that correct specification of noise is important to draw correct conclusions, noise itself is generally not treated as worthy of study.

This simplification obscures that noise can be informative and that by exploring the source and nature of noise, one can often draw additional conclusions about the underlying psychological processes because different psychological processes will imply different sources of noise. In this perspective, noise can be a key component of the phenomenon that is being modeled, and exploring noise can therefore shed new light on the nature of the phenomenon. Fortunately, this perspective is recently gaining more traction; researchers are increasingly demonstrating that treating noise as a source of information in itself can lead to breakthroughs in the understanding of psychological processes and, by extension, human behavior. This special section is dedicated to examples of such findings.

In this introductory article, we first provide a historical review of how psychological science has traditionally conceived of noise in psychological research, including some important exceptions from the prevailing perception of noise as nuisance that lead the way toward treating noise as an integral part of the psychological models. The other contributions for this special section provide additional in-depth reviews of some of the themes covered in our historical review. As an illustrative primer, we discuss our own research on the precise/not precise (PNP) model, showing how the study of human intuition and analysis can be enrichened when considering the source and nature of noise. This model uses a mixture distribution to delineate deterministic (precise) and noisy (not precise) responses in data, consistent with Egon Brunswik’s (1956) original conceptualization of analytic and intuitive processes (Sundh et al., 2021). We conclude by briefly introducing the other contributions in the special section.

## A Note on Terminology

With “noise,” we refer to observed variability in the data that is nondeterministic in the sense that the data have to be described by probability distributions. We have endeavored to, when referring to specific types of noise or to concepts that are not strictly speaking noise at all, instead use the appropriate specific term (e.g., variance, heterogeneity, uncertainty, etc.) and, when using the term as a descriptor (i.e., “noisy”), specify the manner by which it applies to the subject.

Note that our definition is intentionally somewhat broader than the popular conception of noise as specifically undesirable variability (e.g., Kahneman et al., 2021). Needless to say, whether noise is undesirable depends on your perspective (for a discussion, see Sanborn et al., 2024 in the present issue), making this conception simultaneously restrictive and ambiguous. Furthermore, whether noise is considered undesirable has little to do with whether it is informative, which is the more important question.

## Exogenous Versus Endogenous Treatment of Noise in Psychological Research

Observable noise distributions can be addressed in two different ways in theoretical analyses and modeling. With an exogenous treatment of noise—by far the most common approach in statistical modeling of psychological phenomena—noise is not assigned an explicit psychological interpretation but is included only as an “in blanco check” to account for whatever residual variance there is in the data. In this case, noise is interesting from only two points of view: First, one wants to minimize noise to maximize the signal-to-noise ratio, in which the signal is usually thought to represent the psychological process. Second, the mathematical properties of noise have to be correctly specified; otherwise, the estimated parameters of the model can become biased and misleading. Consequently, noise in itself is of little or no scientific interest.

**Fig. 1.**
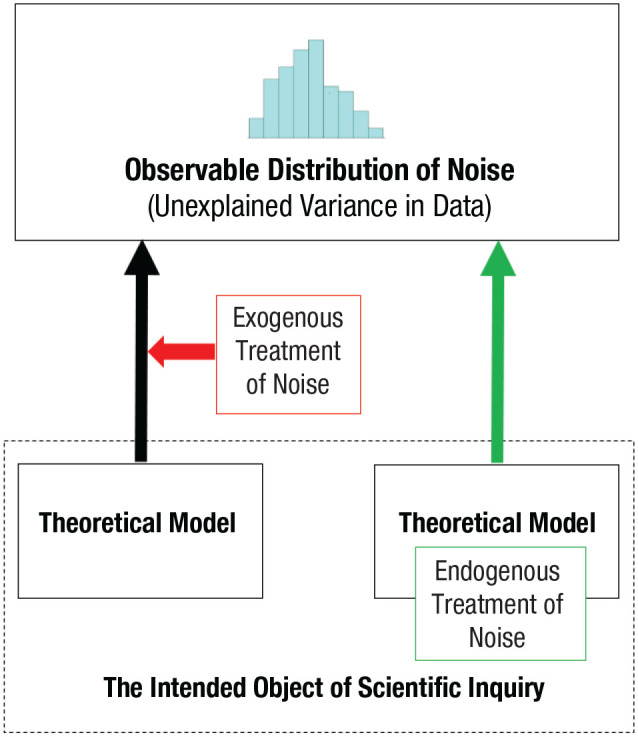
Two ways to treat the noise in the scientific inquiry. (Left) The origin of noise is treated as exogenous to the psychological model, a nuisance to be controlled for in the identification of the process. In this case, there is little scientific interest in understanding the origins of the noise. (Right) The noise is treated as endogenous to this psychological model, part of the explanandum, and informative about the nature of the process.

By contrast, with an endogenous treatment of noise, noise is regarded as part of the explanatory model and therefore part of the object of the scientific inquiry. This means that the properties of noise can be informative about the psychological processes (see the section below on the PNP model). It also means that one can consider the potential functional consequences of this noise, including the possibility that it may sometimes serve adaptive purposes. In this case, probability distributions are an expected property of the data that derive from the psychological process itself, capturing the effects of the extant but at the time of inquiry typically unknown states of the underlying perceptual, cognitive, or neural processes. These probability distributions are therefore diagnostic and informative. See Figure 1 for a schematic illustration of this principle.

## Noise in Psychological Research: Past to Present

As old as the concept of measuring mental processes is the notion that some observations from such measurements include noise (Clayton & Frey, 1997). This was recognized by Fechner (1860/1996) and Thurstone (1927) in their psychophysical studies and by Ebbinghaus (1885/1913) in his studies on memory. The basic thought was that an observed score was made up of, on the one hand, a true score and, on the other hand, noise arising because of uncontrolled independent influences (Boring, 1950; Wixted, 2020). In this perspective, noise could stem from numerous sources (Cox, 1961; Pitt et al., 2002; Wixted, 2020), such as imprecision of measurement tools, variability between individuals, and variance in the sensory system during the encoding and processing of stimuli (i.e., neural noise).

Inspired by the work of Laplace and Gauss, noise was assumed to be normally distributed and came to be seen as a nuisance that needed to be accounted for or eliminated to improve statistical power (Clayton & Frey, 1997; Cronbach, 1957). Put differently, the idea was to separate information-bearing patterns from random (and thus noninformative) patterns (Green & Swets, 1966; Macmillan & Creelman, 2004; Wixted, 2020).^2^ To this day, the pursuit to control for noise exogenous to the model is reflected in the various procedural and statistical methods researchers use (recurrent in psychology textbooks for students; e.g., Field, 2013; Pedhazur & Schmelkin, 2013; Shadish et al., 2002; Tabachnick et al., 2007). It has also been subject of discussion in popular-science discourse aimed at the public on how to improve predictions and judgments (Kahneman et al., 2021; Silver, 2012).

Luce (1995) observed that the way noise is treated in psychological research, in concert with the way psychological phenomena are modeled, effectively limits the precision of conclusions in psychology compared with other fields. In Luce’s perspective, the magnitude of nonsystematic errors could be reduced to close to zero in other fields, but he was pessimistic toward achieving this in psychology, in which the very object of study itself appeared to be a source of error. However, although most psychological research in the 19th and the 20th centuries focused on how to reduce and control for noise, some other scientific fields took a different route. Perhaps the most prominent example is situated in evolutionary theory (Darwin, 1859), in which variation in individuals’ genetic makeup is essential for the development and adaption of species and thus a fundamental component of the theory (Calvin, 1987; Gregory, 2009). Another example is the exploding interest across scientific disciplines (e.g., physics, biology, mathematics) in stochastic processes that came as a result from Einstein’s (1905) model of Brownian motion (Molenaar, 2004).

Although the study of noise has generally received relatively little interest among psychologists, there have been notable exceptions, seemingly of two types: methodological, in which new tools are developed to provide a more complete description of noise as exogenous to the model, and conceptual, in which these methods are used to provide a description of noise as endogenous to the model. Naturally, methodological and conceptual advances are often intertwined, sometimes even within the same study; empirical observations that do not fit into the traditional framework make methodological advances necessary, and methodological advances, in turn, open up new venues for empirical research. Indeed, many prominent theories in psychology are grounded in the development of inference statistics (e.g., signal-detection theory and attribution theory; Gigerenzer & Murray, 2015).

As described in the next section, one methodological theme questions the traditional practice of assuming that measurement errors are (always) normally distributed, opening for the possibility that other distributions, such as the binomial or log-normal distributions, might be necessary. A more conceptual theme focuses on the notion that deterministic models with an additive error term are often insufficient to model noise. Finally, a third theme, associated with both the methodological and conceptual contexts, focuses on how different sources of noise (e.g., between-subjects variations, meaningful inter- and intraindividual differences, noise in the environment vs. within the organism itself, and levels of aggregating individual responses) can be decomposed into measurable counterparts.

### Non-Gaussian error distributions

More than 50 years ago, John Augustus Keats (1967) proclaimed in the *Annual Review of Psychology* that “the important assumptions really concern the error process and the key assumption of the homogeneity of the error process seems to the reviewer to be the most questionable in practice” (p. 226). Others have raised similar concerns (Bock & Wood, 1971; Kristof, 1963; Luce, 1977, 1995), also with seemingly little effect on the field at large. Researchers attempting to highlight the possibility of using the distribution of errors as identification markers for specific mental processes (e.g., intuition and analysis: Brunswik, 1956; Hammond et al., 1983) have been similarly overlooked.

Recently, there are promising signs that the tide is turning, with researchers examining how departures from the convenient Gaussian distribution can be useful for modeling and understanding psychological processes (Aquino et al., 2018; Fengler et al., 2021; Michell, 2020; Oberauer, 2023; Sundh et al., 2021; Tomić & Bays, 2022; Wilson et al., 2021; Zhou et al., 2021). For example, Wilson et al. (2021) showed that different statistical distributions can be used to model different cognitive processes in explore-exploit decisions, Aquino et al. (2018) discussed how deviations from Gaussian distributions can be used to model how time intervals are internally represented, and Fengler et al. (2021) illustrated how non-Gaussian assumptions can provide new pathways for modeling decision-making behavior in the realm of neural-network-instantiated drift-diffusion models.

### Probabilistic models

A standard assumption in many theoretical and statistical models is that the process is deterministic, with any unexplained variation in data attributed to irrelevant (exogenous) factors. This is adequate in some situations, but often, it is more accurate to describe noise as the consequence of a psychological process that is in itself probabilistic, in which case, noise is endogenous and potentially diagnostic of the process in question (Atkinson et al., 1965; Becker et al., 1963; Bower & Trabasso, 1964; Iverson, 1991; Luce, 1995; Regenwetter et al., 2010). Put differently, whereas traditional application of probability theory was concerned with controlling exogenous noise, the movement described here turned to using probability theory in the perspective that chance is part of the phenomenon under investigation (Gigerenzer & Murray, 2015).

This idea also occurs, for example, in the area of economic decision-making. The standard normative framework, expected-utility theory (von Neumann & Morgenstern, 1944/2007), is deterministic in the sense that it implies that people consistently choose the same course of action in similar situations. On the other hand, a substantial amount of research has shown that people’s choices are stochastic (e.g., Blavatskyy, 2007; Blavatskyy & Pogrebna, 2010; Hey, 2005; Hey & Orme, 1994; Luce, 1959; Regenwetter & Davis-Stober, 2018; Regenwetter et al., 2010; Webb, 2019). Probabilistic models take this stochasticity into consideration, with different potential sources of the observed variation. For example, in the case in which a decision maker chooses between two prospects that both involve two valued outcomes with probabilities associated with each, it could be argued that although the process itself is essentially deterministic, the interpretation of the probabilities and values of the prospects are themselves stochastic (e.g., Loomes & Sugden, 1995). For example, it is not necessarily the case that an individual perceives a probability of 0.50 or an outcome of $100 exactly the same way on every occasion. Alternatively, the stochastic component might enter the decision-making process when individuals execute their deterministic preferences (Blavatskyy, 2007). That is, the decision maker does indeed weight exactly $100 according to a probability of exactly 0.50, but stochastic processes affect the reporting of this deterministic preference. The contribution by Regenwetter et al. (2024) in the present issue highlights this perspective in the realm of moral judgment, in which they compare models that model behavioral variability as a fixed transitive preference perturbed by response noise or as a probabilistic mix of transitive-preference orders.

Another instance of including noise as endogenous to the model is provided by random-walk models, which were developed specifically for understanding how stochastic processes can be modeled as dynamic real-time processes (e.g., Forstmann et al., 2016; Stone, 1960). One instantiation of such models is sequential-sampling models, which propose that people gradually accumulate information until a threshold of evidence is reached. The application of sequential-sampling models (Busemeyer, 1985; Ratcliff et al., 2016) has become increasingly popular, arguably because of their ability to explain a large variety of phenomena (e.g., Dror et al., 1999; Krajbich, 2019; Liesefeld & Janczyk, 2019; Pedersen et al., 2017; Trueblood et al., 2014; Zilker & Pachur, 2022) and ground explanations in neurocognitive theory (Forstmann et al., 2016; Mittner et al., 2016; Turner et al., 2017). The most influential sequential-sampling model, the drift-diffusion model (Ratcliff et al., 2016), explains the mechanisms that give rise to accuracy and reaction-time distributions in choice tasks and parameterizes these mechanisms in a manner that has a clear psychological meaning. Extracting parameter estimates from individuals can then be used to, for example, understand how different groups differ from each other. For instance, Lawlor et al. (2020) applied the drift-diffusion model to a clinical setting and showed that depressed adults accumulated the evidence needed to make decisions more slowly than control participants did, insights that open up new possible causal pathways for explaining depression.

Relatedly, sampling-based models of judgment (Chater et al., 2020; Sanborn & Chater, 2016; see also Sanborn et al., 2024, in the present issue) and decision-making (Stewart et al., 2006; Stewart & Simpson, 2008) have recently proven very successful at explaining a range of behaviors and biases. Such models naturally explain the stochasticity of judgment and decision-making as a consequence of being based on a limited number of sampled instances. For example, consider the phenomena of probability matching, which is the tendency of people to choose alternatives with stochastic payoffs in proportion to the probability of payoff rather than simply sticking with the alternative with the highest probability of payoff (e.g., Koehler & James, 2009). If these decisions are based on small random samples of singular past experiences that are stochastically activated by the context, then probability matching is an unavoidable consequence (e.g., if only one sample is retrieved, the responses will exactly match the underlying probability distributions).

### Decomposition of sources

If noise stems from several different sources, the failure to identify these makes it hard to disentangle noise exogenous to the model from noise endogenous to the model (e.g., Brunswik, 1952; Golding, 1975; Greenwald, 1976; Hammond, 1955; Riker, 1995; Zhu et al., 2022). One seminal illustration of how the noise in judgment can be decomposed is with the lens-model analysis developed in the neo-Brunswikian tradition to judgment and decision research (Hammond & Stewart, 2001). With the lens-model equation, the observable noise in judgment—conceived of as the deviations between the judgment and the criterion (true state)—can be decomposed into, on the one hand, noise as irreducible consequences of the merely correlative relations between the available cues and the criterion and, on the other hand, noise arising in the judgment process itself because of the inconsistent (noisy) use of the cues. Two important contributions by the lens-model analyses are to emphasize that judgments are often constrained by the inherent predictability allowed by the available cues and to point out that inconsistency (nonsystematic noise) is typically a more important constraint than cognitive bias on the quality of human judgments (later emphasized also by Kahneman et al., 2021).

Juslin and Olsson (1997) also showed that it is possible to discriminate between Thurstonian and Brunswikian uncertainty (“uncertainty” being translatable to “noise” for the present context) on empirical grounds in the context of a psychophysical discrimination task. Whereas Thurstonian uncertainty implies that decisions made by participants who are given the same discrimination task are stochastically independent or uncorrelated (i.e., response independence), Brunswikian uncertainty is the exact reverse. In the latter case, participants have adapted to the same environment and have developed similar cue structures. As a result, decisions made by different participants in response to the same task are dependent on or linked with one another (i.e., response dependence). Each type of uncertainty predicts different relationships between confidence, stimulus difficulty, and response times—qualitative elements of data that can be obtained experimentally and compared. Other historical examples can be found in the areas of learning and categorization (Briggs et al., 1957; Clayton & Frey, 1997), cognitive organization (Binder & Wolin, 1964), motor behavior (Slifkin & Newell, 1999), semantics (Jones & Thurstone, 1955), and clinical assessment (Einhorn, 1986; Meehl, 1954; Thomas, 1973) and in the “Murphy decomposition” of the Brier score used to analyze validity of probabilistic forecast (Murphy, 1973).

The pursuit to decompose noise into meaningful components recently surfaced across several research topics in psychology: decision-making (e.g., Bhui et al., 2021; Birnbaum & Quispe-Torreblanca, 2018; Cavagnaro & Davis-Stober, 2014; Regenwetter & Robinson, 2017), forecasting (e.g., Satopää et al., 2021), attention (e.g., Luzardo & Yeshurun, 2021), and fear extinction (e.g., Lonsdorf & Merz, 2017). Kahneman et al. (2021) provided a recent and influential example of this research track and argued that there can be multiple reasons for why noise arises, from cognitive biases to differences in skill, emotional reactions, and group dynamics. In the present issue, Sanborn et al. (2024) and Seitz et al. (2024) provide additional examples.

There have also been efforts to understand how different types of noise can be attributed to biological structures (e.g., Kanai & Rees, 2011), how researchers can design experiments with different sources of noise in mind (Myung et al., 2013; Yang & Molano-Mazón, 2021), and whether there can be adaptive benefits of different sources of noise (e.g., Findling & Wyart, 2021; Sternad, 2018; Zhu et al., 2020). For example, Sternad (2018) illustrated how noise in the complex human neuromotor systems can be beneficial when exploring solutions for novel tasks.

## Illustrative Primer: The PNP Model

In this article, we argue that in research in psychological science, noise in human behavior is usually treated as exogenous to the model of the psychological phenomenon under study. Typically, the phenomenon is described using a deterministic statistical model with an additive (typically Gaussian) exogenous error term. In this perspective, noise is only of interest insofar as it needs to be minimized and correctly specified to obtain unbiased estimates of the parameters of the underlying deterministic model. For individuals who know where to look, however, noise can itself be a source of information. All noise has a source, and different sources of noise can give rise to different expressions of noise. It follows that if these different sources of noise are associated with distinct processes, then one can use the way noise is expressed in data to draw conclusions regarding those processes.

In the following, we provide an extended illustration using a computational-modeling framework—the PNP model—developed for the exact purposes of empirically determining how different sources of noise are associated with distinct psychological processes. Therefore, the PNP model is an example of an approach consisting of the following three steps:

1. Articulate the psychological mechanisms that are to be investigated.
2. Determine the properties of the noise expected to arise in this process (e.g., binomial, multinomial, or continuous; expected distribution shape).
3. Derive and test empirical predictions.

Together with the additional articles provided in this special section, we hope this illustrative primer will provide enough practical detail to serve as inspiration for the broader community of psychological researchers to turn the theoretical promise of treating noise as information into actual practice.

**Fig. 2.**
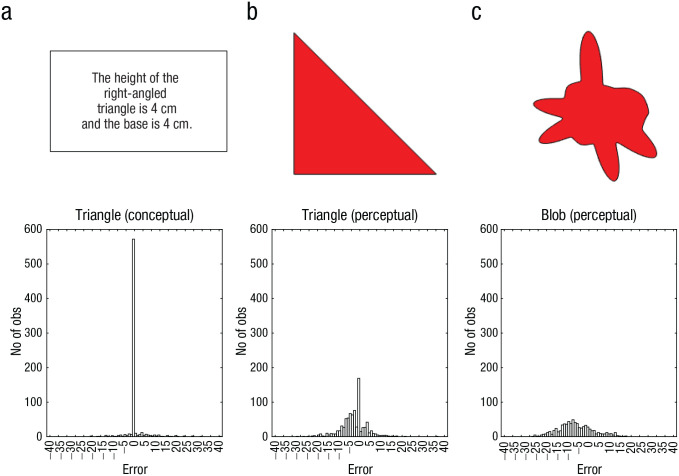
Examples of the stimuli used in Experiment 1 in Sundh et al. (2021). (a) The condition with a conceptual triangle, (b) the condition with a visual triangle, and (c) the condition with a blob shape. In all conditions, the participants were asked to estimate the area covered by the object in square centimeters (cm^2^). Below each stimulus is the distribution of deviations from the criterion value in the corresponding condition, with a distinctly leptokurtic distribution for the conceptual triangle, a (roughly) Gaussian distribution for the blob shape, and an intermediate result for the perceptual triangle. The figures are reproduced from Sundh, J., Collsiöö, A., Millroth, P., & Juslin, P. (2021). Precise/not precise (PNP): A Brunswikian model that uses judgment error distributions to identify cognitive processes. *Psychonomic Bulletin & Review, 28*(2), 351–373.

The PNP model (Sundh et al., 2021) exemplifies the principles discussed in the previous section applied in the context of cognitive psychology. The model takes inspiration from the results observed by Brunswik (1956), in which judgments in perceptual tasks produced a Gaussian error distribution and mental arithmetic produced a leptokurtic error distribution centered on the correct response (for an illustration of these distributions shapes, see Fig. 2). Brunswik used these results as the basis of a definition of intuitive and analytic processes, in which intuition is defined by approximation (i.e., a Gaussian error distribution) and analysis is defined by precision (i.e., a leptokurtic error distribution), long before these concepts became synonymous with dual-process theories (Evans, 2008; Evans & Stanovich, 2013).

In line with Brunswik’s ideas, it is natural to assume that intuition draws primarily on the parallel and distributed processes that are involved in perception and memory, which typically engages many independently operating units (e.g., neurons). As illustrated by the famous Galton board (Galton, 1894), the “vote” across many independent units (events) produces a Gaussian distribution. By contrast, analysis primarily involves deterministic application of rules to symbols. When successfully applied, these latter procedures invariably produce the same exact answer, but if there is an occasional imperfection in the execution of these processes, the error may sometimes be large. As noted by Brunswik, this suggests a leptokurtic distribution, in which most of the answers are exactly correct but with occasional (potentially large) errors.

This exemplifies the first two steps in the previous section: articulation of the psychological mechanisms and processes involved and derivation of the likely properties of the noise that can arise in the processes. Because of its operational nature, Brunswik’s definition can be readily observed by analyzing the response distributions; in practice, using the relative proportion of precise responses relative to responses perturbed by error.^3^ This illustrates the third step discussed in the previous section: Derive and test empirical predictions.

The PNP model instantiates this idea using computational cognitive modeling, separating the responses that precisely align with some deterministic process from those that are perturbed by error. In this sense, the PNP model does not refer to one single computational model because it need not be associated with any particular cognitive process. Rather, it is a framework through which computational models of cognitive processes can be expressed and that ultimately may be applicable beyond the Brunswikian conception of intuition and analysis. However, for present purposes, we focus on how the PNP model is used to study intuitive and analytical processes (because that was the initial purpose of the model). We further discuss what implications this carries for dual-process theories of intuition and analysis and how the model can be used to delineate different memory processes.

### Noise in intuitive and analytical processes

In the PNP model, the proportion of error-perturbed responses is measured by the parameter λ. Therefore, if we conceptualize the presence or absence of error for each response as the outcome of a binomial variable *B*, where the outcome *b* = 1 denotes error and *b* = 0 denotes no error, and given that *P*(*b* = 1) = λ and *P*(*b* = 0) = (1 – λ), we can express the PNP model as

(1) $$y|\left(B=b\right)=\{\begin{array}{c} g\left(x|\theta \right)+N\left(0,\sigma^{2}\right),b=1\\ g\left(x|\theta \right),\;\;\;\;\;\;\;\;\;\;\;\;\;\;b=0 \end{array},$$

where *g* is a function representing some psychological process (e.g., a cognitive algorithm), **x** is a vector of input variables, **θ** is a vector of parameters, and *y* is the predicted response. An intuitive process would therefore result in a high value of λ, and an analytic process would result in a low value of λ, thereby contributing a way that the degree of intuition and analysis, from a Brunswikian perspective, can be measured directly from the data. Noise is thus endogenous to the model in the sense that the pattern of errors is assumed to reflect the nature of the underlying cognitive processes (intuitive or analytic).

In Sundh et al. (2021), we applied the PNP model to data from three different experiments. The purpose of Experiment 1 was first, to replicate Brunswik’s results by contrasting perception with mental arithmetic and second, to extend the paradigm with a task that involved the potential for both arithmetic and perception. To this end, we used three tasks illustrated in Figure 2, in which the participants estimated the area of a geometric shape: Figure 2a illustrate the estimation of the area of a right-angled triangle from numerical measurements of the base and height (mental arithmetic), Figure 2b illustrate the estimation of the area of a right-angled triangle from a visual representation (perception and mental arithmetic), and Figure 2c illustrate the estimation of the area of an irregular blob from a visual representation (perception).

The results were consistent with Brunswik in that the response distribution for the numeric task was strikingly leptokurtic and the response distributions in the two visual tasks were Gaussian, with a more pronounced peak in the visual triangle condition. The task that involved mental arithmetic thus disclosed a high frequency of perfectly correct answers, marred by occasional errors, yielding the leptokurtic distribution that is the hallmark of analytical cognitive processes. The task that required direct perceptual assessment, the perceptual blob, produced a (roughly) Gaussian distribution, the signature of an intuitive cognitive process.

The reasons for the intermediate result, with a roughly Gaussian distribution but a spike at the correct value, for the perceptual triangle condition are interesting. The perceptual encoding of the base and height would suggest perceptual (intuitive) noise in both of these inputs, which, in turn, would suggest that also after the participants have applied their mental arithmetic to these inputs, the output should reveal the Gaussian distribution typical of intuitive cognitive processes. This is indeed observed for most of the responses. By design of the task, however, the base and height produced on the screen for the stimuli were single digits. Because participants typically rounded their percepts to single digits before executing their mental arithmetic, for these specific stimuli, the responses coincide exactly with the correct answers.

Applying the PNP model separately to individual participant data confirmed these results in terms of intuitive and analytic processes; for the numeric triangle, the median λ was .05; for the visual triangle, the median λ was .86; and for the visual blob, the median λ was .92. We can therefore conclude that the results are consistent with Brunswik’s hypotheses and that the PNP model successfully identifies these Brunswikian instantiations of intuition and analysis.

To confirm that the principles are broadly applicable in cognition, it is necessary to demonstrate that both intuitive and analytical processes are also present in symbolic tasks that involve no perceptual noise. In Experiment 2 of Sundh et al. (2021), we therefore extended the analysis to a number of cognitive tasks with the same mathematical structure but different content.

Let us consider the example of a willingness-to-pay (WTP) task, in which participants assess what they would be willing to pay to participate in a basic lottery, with a probability *p* to gain a certain monetary reward *v*, otherwise nothing. In this case, the expected value (EV) of the lottery is estimated by multiplying the probability of the monetary reward with the value of the reward, so that EV = *p* × *v*. However, it is arguably rational (although not necessary) to pay less than the EV because otherwise, you would not, on average, gain anything from your participation. We can simulate this by adding an intercept and a weight parameter to the EV, giving us the simple model WTP = *α* + β(EV). We can then fit this model to participant data within the PNP framework by defining *g*(**x**|**θ**) = *α* + β(EV), simultaneously fitting, on the one hand, *α* and β as part of the cognitive algorithm described above, and, on the other hand, λ as part of the statistical expression of the PNP model (see Equation 1).

**Fig. 3.**
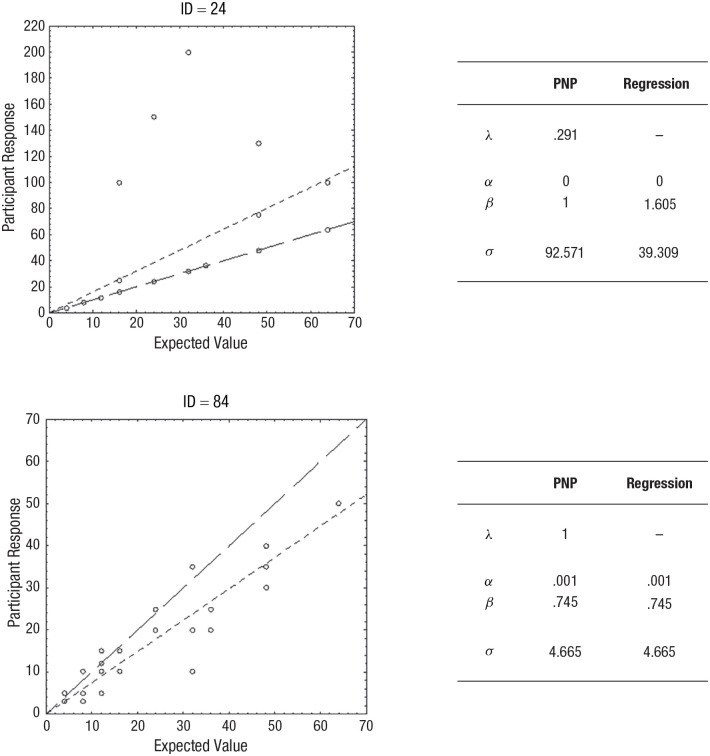
Examples of results in Experiment 2 in Sundh et al. (2021). The responses by two participants in a willingness-to-pay task, with estimated parameter values from the precise/not precise (PNP) model and a conventional regression model. A line representing the predictions of the regression model (small-dashed line) and a reference line *x* = *y* (large-dashed line) is included. In the upper panel (ID = 24), the predictions by the PNP model coincide with the reference line, and in the lower panel (ID = 84), the predictions of the PNP model coincide with the predictions of the regression model. Note that some data points overlap. The figures are reproduced from Sundh, J., Collsiöö, A., Millroth, P., & Juslin, P. (2021). Precise/not precise (PNP): A Brunswikian model that uses judgment error distributions to identify cognitive processes. *Psychonomic Bulletin & Review, 28*(2), 351–373.

In Sundh et al. (2021), we performed the exact modeling described above using empirical data. An interesting pattern emerged, signifying that participants tended to approach the task in one of two ways: either by a noisy and approximate process or by using precise calculation with occasional errors. For two examples of such participants, see Figure 3. It is apparent that participant ID = 24 calculated the exact EV (an analytic process), whereas participant ID = 84 produced estimates that were generally lower than the EV by a more approximate method (an intuitive process). Note that for participant ID = 24, the parameters identified by the PNP model implies that most of the responses (i.e., 1 – λ = .78, or 78%) are exact computations of the EV of the gamble. For analytic processes, an ordinary regression will not capture this systematicity, but the PNP model will, whereas for an intuitive process, the PNP model and the ordinary regression will naturally coincide.

### Noise in the dual-process view of cognitive biases

The Brunswikian perspective differs in many ways to the dual-processes perspective (e.g., Evans & Stanovich, 2013). Dual-process theories often suggest an interplay in which quick, unconscious, and often relatively feeble intuitive processes are supervised by conscious, analytic processes that comprise people’s rational competencies (De Neys & Pennycook, 2019; Evans & Stanovich, 2013; Sloman, 1996). Within the dual-processes perspective, the main criteria for identifying intuitive and analytic processes have been in terms of the speed, autonomicity, and control of the cognitive processes rather than empirical error distributions. The dual-processes perspective therefore implies that intuitive processes can potentially be conceptualized as either noisy or noise-free.

As an illustration, consider the bat-and-ball problem:

A bat and a ball cost $1.10. The bat costs $1.00 more than the ball.

How much does the ball cost? ____ cents

**Fig. 4.**
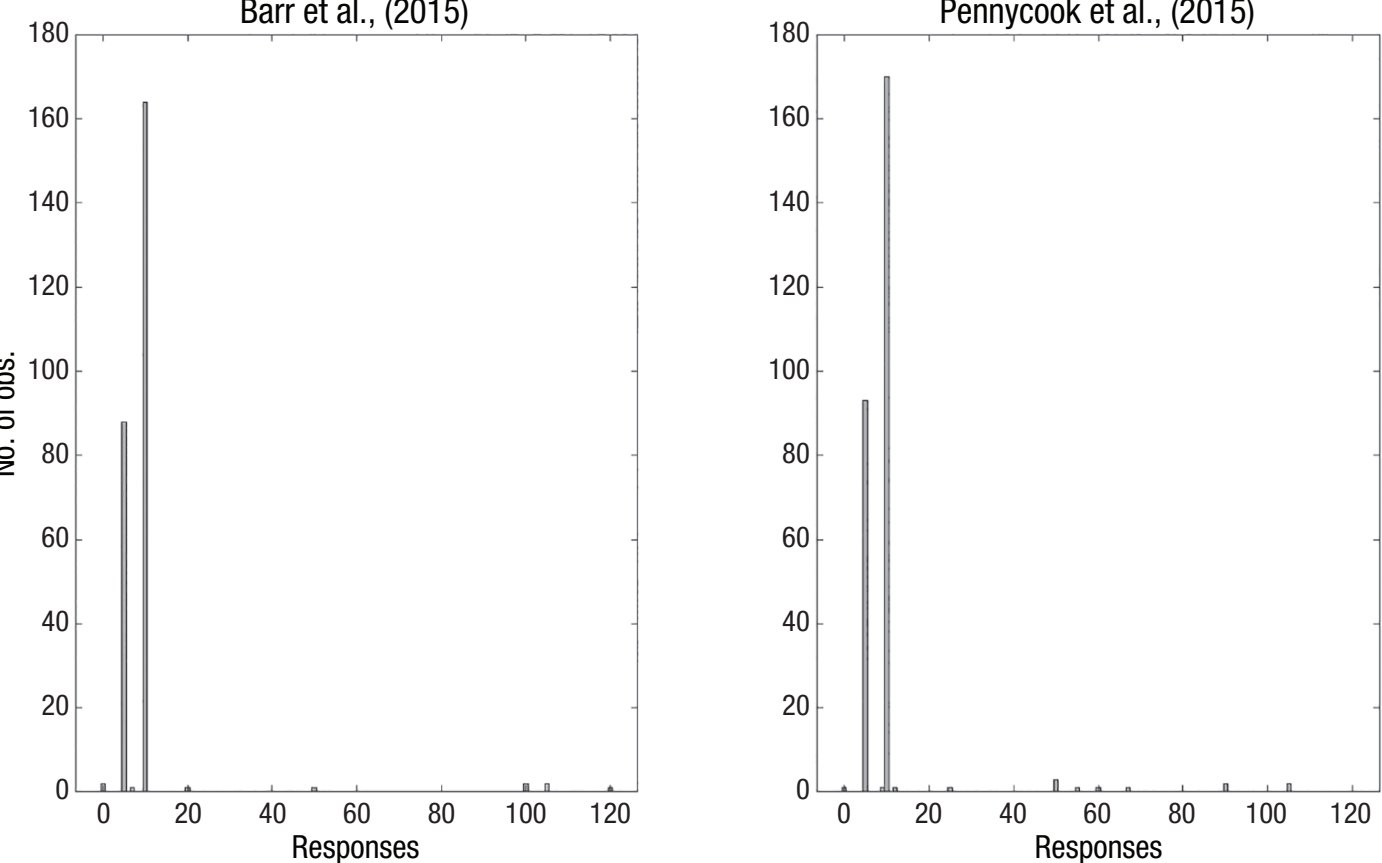
The distribution of responses to the bat-and-ball problem from Experiment 1 in (left) Barr et al. (2015) and (right) Pennycook et al. (2015). The data from Pennycook et al. contain two outlier responses of 210, which were excluded to improve readability.

The correct answer is 5 cents, but the most common answer is 10 cents. The dual-process perspective emphasizes that the typical answer of 10 cents derives from an intuitive process that remains unchecked by the appropriate analytical insights (Frederick, 2005). By contrast, a Brunswikian approach suggests that this is an oversimplification. Figure 4 illustrates the response distributions from two well-known published experiments with the bat-and-ball problem. In Pennycook et al. (2015), 94.3% gave either the incorrect 10-cents answer (60.1%) or the correct 5-cents answer (33.3%). In Barr et al. (2015), 96.2% gave either the incorrect 10-cents answer (62.6%) or the correct 5-cents answer (33.6%). Considered from the Brunswikian perspective, neither the 5-cent nor the 10-cent response disclose anything like the Gaussian distributions that identify an intuitive estimation—the distribution is rather strongly bimodal, with spikes at the two modal responses. This suggests that responses derive from a competition between two deterministic (i.e., analytic) algorithms: One algorithm that is simple to execute but wrong (corresponding to *x* + 1.00 = 1.10 and solve for *x*) and one algorithm that is more complex but correct (corresponding to *x* + (*x* + 1.00) = 1.10 and solve for *x*; Lauridsen et al., 2024).

Without considering noise as endogenous to the model, we would not be able to identify that both participants that respond correctly and those participants that respond incorrectly to the bat-and-ball problem rely on equally deterministic cognitive processes, suggesting that both entail explicit calculation of an algorithm (i.e., analytic processing) rather than one analytic and one intuitive process.

### Noise in memory processes

Because the PNP model can be used with a variety of functions, it is possible to apply this operational definition also to memory and thus disentangle memory-based processes that are more or less affected by noise. Application of the PNP model to memory data suggests the use of memory in two different ways: first, by recall of exact values in a largely deterministic way that yields leptokurtic response distributions (e.g., when you recall the year of your birth) and second, by similarity-based inference based on retrieval of previously encountered objects that tends to yield Gaussian distributions (e.g., when you guess the age of an unknown university professor based on other professors whose age you know). When treating noise as endogenous to the memory-based model, we thus gain important information that allows us to differentiate between reliance on rote memory versus similarity-based inference (for further discussion, see Collsiöö et al., 2023).

The generalized-context model (GCM) of category learning (Nosofsky, 1986, 2015) has frequently been applied to understand also how memory is used in multiple cue judgments that refer to a continuous criterion (e.g., Juslin et al., 2003). We have used the PNP model to identify two different uses of memory in multiple-cue judgment, into either precise (intuitive) or nonprecise (analytic) reliance on exemplar-based memory (see Collsiöö et al., 2023). Good fit by the GCM has typically been interpreted in terms of automatic similarity-based memory processes, recall of previous exemplars, and informal integration across these exemplars. This suggests an approximate process that is subject to Gaussian random noise (intuition). However, it is also possible that an individual would engage exemplar memory in a deterministic analytic fashion, as when you recall an overlearned fact.

In Collsiöö et al. (2023), we illustrated how the original GCM can naturally parameterize both similarity-based memory processes and rote-memory processes, and we applied the PNP model to previously collected multiple cue judgment data that are well accounted for by the GCM. Surprisingly, given the standard interpretations of the results in the literature on categorization and multiple cue judgment (see e.g., Juslin et al., 2003, 2008; Nosofsky, 2015; Nosofsky & Johansen, 2000), the PNP model indicates that the good fit of the GCM on individual participants data is often the effect of a deterministic memory processes involving rote memorization of exemplars rather than the approximate intuitive similarity-based inference often assumed^4^ (for similar results based on other methods, see Bröder et al., 2017; Izydorczyk & Bröder, 2023). This highlights how treating the noise in the cognitive process as endogenous to the model results in qualitatively different conclusions in contrast to previous research in which noise has been treated as exogenous to the model (e.g., Juslin et al., 2003).

If replicated, these results could be problematic for conclusions that equate good fit by the GCM with reliance on automatic similarity-based inference. Many previous studies have relied on a small set of repeated training exemplars (e.g., Juslin et al., 2003), designs that might invite rote memorization of a few individual and often-repeated exemplars in ways that may not generalize to more complex real-life tasks.

## Discussion

In this introductory article on the role of noise in psychological research, we have distinguished between an exogenous and an endogenous treatment of noise in data. The by-far most common approach is to treat noise as exogenous to the theoretical and statistical model of the phenomenon in question, in which it is essentially viewed as a noninformative nuisance. In this perspective, noise should be minimized and correctly specified so as not to bias the estimation of the underlying deterministic process that is the object of study. By contrast, treating noise as endogenous to the model means that noise is both diagnostic of the nature of the processes and of potential (positive or negative) functional significance.

In a historical review, we briefly traced the role of noise in psychological research, including discussions of previous endogenous treatments of noise in the literature. From the review, it is clear that endogenous treatments of noise can take many shapes and forms and are of relevance across the psychological subdisciplines. Furthermore, it illustrates that theoretical advances are often the consequences of developments in methods and technology (Gigerenzer, 1991; Gigerenzer & Murray, 2015). It is likely no coincidence that the advances in several fields over the last decade (i.e., the computational revolution; Hofman et al., 2021) have allowed for a new breed of computational models in which noise can enter as a natural component to be formalized and empirically tested.

Finally, we used our research on the PNP model as an example of the endogenous treatment of noise in cognitive modeling (see Collsiöö et al., 2023; Sundh et al., 2021; Lauridsen et al., 2024). Here, we illustrated that the PNP model supports and recovers the intuitive and analytical processes originally discussed by Brunswik (1956) but also that it can be applied to “purely” symbolic tasks that involve no (functionally relevant) perceptual noise. We also reviewed how the PNP model extends the distinction between intuition and analysis in judgments based on memory processes and how the Brunswikian perspective and the PNP model relate to the dual-system perspective and can afford new ways to approach the role of intuition and analysis in the context of cognitive biases.

The rest of this special section provides a wider sample of examples of how the endogenous treatment of noise can enrich the understanding of cognitive processes. Regenwetter et al. (2024) compare models of behavioral variability as a fixed transitive preference perturbed by response noise or as a probabilistic mix of transitive preference orders. They concluded that moral preferences are transitive but uncertain (i.e., noisy), in the sense that moral judgments are noisy instantiations of transitive preferences. These results indicate that there is, as they put it, “order underlying the apparent chaos” and that individuals’ seemingly idiosyncratic choices should therefore not be interpreted as fundamental irrationality or as disregard for moral principles.

Sanborn et al. (2024) examine noise in perceptual and preferential judgments, finding that contrary to popular interpretations, the major contributing factor to noisy behavior may be in the cognitive computations rather than in the sensory and motor systems. In this perspective, the particular characteristics of behavioral noise in these contexts, that is, heavy tails and long-range autocorrelations, are hallmarks of sophisticated sampling algorithms. Consequently, noise in human behavior aids exploration of hypotheses and should arguably not be considered a bug as much as a feature.

Seitz et al. (2024) review theories of categorization and their implications regarding the nature of intraindividual noise across trials based on the different categorization mechanisms implied by different theories. They demonstrate that noise in either perceptual intake, cognitive processing, or response generation can express itself in different ways and therefore can lead to different empirical response distributions. Thus, investigating intraindividual noise and interindividual noise can allow one to draw conclusions that would otherwise not have been possible.

We believe that all of these contributions, in addition to being important contributions to psychological research in their own right, represent excellent examples of the possibilities inherent in using noise to inform scientific inquiry by treating it as a source of information. Doubtless, there are many more possibilities still, and we hope that this special section will inspire our readers to follow up some of these themselves.

## Transparency

*Action Editor:* Rogier Kievit

*Editor:* Interim Editorial Panel

## Notes

1. Usually represented by the parameter ε ≈ *N*(0, σ).

2. We do not claim that all psychological research from this era necessarily treated noise as nuisance. For example, another context for noise is when it is treated neither as a mere nuisance nor as the object of study but as an integral part of, or instrument for, the measurement itself—as in the case of Thurstonian scaling or instantiations of signal-detection theory (Gigerenzer & Murray, 2015). However, it is fair to say that the overwhelming majority of research was indeed focused on treating noise as nuisance.

3. Note that “precise” here denotes responses that are precise according to some psychological process or algorithm, which may or may not coincide with the objective criterion of a given task.

4. Note that compared with the results of Experiment 2 in Sundh et al. (2021), participants best described by the GCM in Collsiöö et al. (2023) were spread along an analytic-intuitive continuum (see Hammond et al., 1983) rather than exclusively confined to purely analytic or intuitive instantiations of the GCM.
